# Time-restricted feeding alleviates arthritis symptoms augmented by high-fat diet

**DOI:** 10.3389/fimmu.2025.1512328

**Published:** 2025-02-13

**Authors:** Zsófia Búr, Bernadett Vendl, Ágnes R. Sűdy, Zalán Lumniczky, Csongor G. Szántó, Attila Mócsai, Krisztina Káldi, Krisztina Ella

**Affiliations:** Department of Physiology, Semmelweis University, Budapest, Hungary

**Keywords:** leukocyte, inflammation, circadian, neutrophil, monocyte, leptin, mouse

## Abstract

Rheumatoid arthritis (RA) affects approximately 1% of the global population. Its hallmark symptoms include severe pain and joint stiffness, which significantly diminish life quality. RA’s development is influenced by multiple factors including unhealthy lifestyle habits. Calorie-rich diets, particularly those high in fat and resulting in obesity, are associated with RA and exacerbate its symptoms. Consequently, dietary modifications are recommended as a complementary treatment. However, adherence is often low due to the restrictive changes required in nutrient composition or caloric intake. Our previous findings indicate that time-restricted feeding (TRF) benefits leukocyte rhythm and mitigates autoimmune responses. In this study we explored the impact of TRF on the severity of K/BxN serum-transfer arthritis (STA) in mice subjected to high-fat diet. Three feeding schedules were implemented: a control (Ctrl) with constant access to standard chow, a high-fat diet group (HF) with *ad libitum* food access, and a high-fat TRF group (HF-TRF) with a 10-hour feeding window during the active phase. After four weeks of conditioning, STA was induced. Although macroscopic markers of inflammation did not differ between the Ctrl and HF groups, histological analysis revealed increased inflammation in HF mice, including expanded edema, pannus formation, bone erosion, elevated synovial neutrophil infiltration and serum leptin levels. Importantly, all these inflammatory markers were significantly reduced in the HF-TRF group, along with synovial IL-1β and monocyte/macrophage counts. Our results indicate that TRF can diminish the impact of a high-fat diet on STA severity, potentially serving as a preventive method and a sustainable therapeutic support for RA management.

## 1 Introduction

Rheumatoid arthritis (RA) is the most common autoimmune disease, which affects approximately 1% of the population worldwide (1). The typical symptoms of this chronic autoinflammatory disease are severe pain and joint stiffness, both of which can impair life quality and emotional state of the patients. The development of RA is affected by several factors, including age, gender, genetic predisposition, hormonal and immunological status, and importantly, lifestyle, which can significantly affect both the onset and the progression of the disease (2, 3). Prevalence of RA is increased under conditions related to chronic stress, regular alcohol consumption and smoking, as well as lack of physical activity (2, 3). Additionally, it is well documented that unhealthy eating habits (*e.g.* a western or high-fat diet) and their consequences – such as obesity and metabolic syndrome – can be risk factors for RA development and significantly exacerbate symptoms of the disease (4). Therefore, in managing RA, dietary strategies are increasingly recognized for their potential to modulate inflammatory processes and alleviate symptoms (3, 5). The Mediterranean diet provides a rich source of omega-3 fatty acids, polyphenols, and other antioxidants, which have been shown to reduce inflammatory markers such as C-reactive protein (CRP) and interleukin-6 (IL-6) (6, 7). Clinical studies have demonstrated that omega-3 fatty acids, predominantly found in fish oil, can significantly reduce joint pain and morning stiffness in RA patients (3, 8). Moreover, the high fiber content in fruits, vegetables, and whole grains promotes a healthy gut microbiota, which may further support the balanced function of the immune system (3, 9). Gluten has been implicated in increasing gut permeability and promoting inflammation (10), and thereby could exacerbate RA symptoms in sensitive individuals. Therefore, a gluten-free diet is also suggested for RA patients, particularly for those with concomitant celiac disease or gluten sensitivity (11). Additionally, reducing the intake of processed foods, red meats, and sugars is advised. Consumption of these foods is associated with increased production of proinflammatory mediators, like advanced glycation end products (AGEs), which can trigger RAGE (receptor for AGEs) signaling and worsen inflammatory responses (12). Emerging evidence also supports the inclusion of certain dietary supplements, such as vitamin D and probiotics, which may have adjunctive benefits in managing RA. Vitamin D has immunomodulatory effects (13), while administration of probiotics may reduce inflammation by modulating cytokine production, improving intestinal barrier function, and enhancing the effects of disease-modifying anti-rheumatic drugs; *e.g.* various strains of Lactobacillus and Bifidobacteria, either individually or in mixed cultures, have shown positive effects on disease activity in RA patients [reviewed in (14, 15)].

Overall, dietary interventions in RA aim to reduce inflammation, manage symptoms, and improve health, highlighting the importance of personalized nutrition in chronic inflammatory conditions. However, the above mentioned methods are mostly based on caloric restriction, changes in nutrient composition or dietary supplement usage, therefore patients’ adherence is often low. Additionally, emerging evidence suggests that timed eating, when restricted to a well-tolerable time interval of the day, might reduce inflammatory processes (16, 17). Timing of nutrient availability is a major input of circadian regulation, which in turn, is known to interact with the pathology of RA. As a sign of this interaction, symptoms of inflammation, such as joint stiffness and pain, typically worsen during the late night and in the early morning (18). It is known that time-restricted feeding (TRF) enhances the circadian clock function in the liver, especially under HF conditions (19). Our previous research indicated that TRF without caloric restriction can strengthen the rhythmic activity of the adipose tissue in mice kept on standard chow diet and mitigate the symptoms of K/BxN serum-transfer arthritis (STA) (20). Moreover, the elevated expression of *leptin*, *tnfα*, *il18* and *nlrp3* in adipose tissue under *ad libitum* conditions compared to TRF indicated a proinflammatory state (20). Leptin production is strongly dependent on nutrient composition as well; in HF fed animals its levels are elevated when compared to standard chow *ad libitum* fed mice (19). Additionally, a high-fat diet induces an increase of IL-1 in adipocytes (19). In RA, IL-1β is a key mediator of the inflammatory response. It promotes the recruitment of immune cells to the synovial membrane and enhances the differentiation and activation of osteoclasts (21). Leptin is also known to promote the activation and migration of myeloid cells through enhancing the expression of the β2-integrin CD11b (22, 23). Additionally, both IL-1β and leptin propagate the proinflammatory characteristics of fibroblast-like synoviocytes.

The aim of the present study was to investigate whether the negative effects of unhealthy eating on arthritis could be counteracted by proper meal timing. Therefore, we investigated how TRF restricted to the first 10 hours of the active period of the day impacts the development of STA in mice kept on a high-fat diet. Our results suggest that TRF can rescue many negative effects of this dietary regimen on arthritis symptoms. For several disease indicators, TRF, although applied alongside a high-fat diet, reduced the level of inflammation even compared to the group consuming a standard diet *ad libitum*. Our data suggest that well-timed food consumption may have a preventive effect on the development of autoimmune disorders.

## 2 Method

### 2.1 Animals and diets

**Figure 1:**
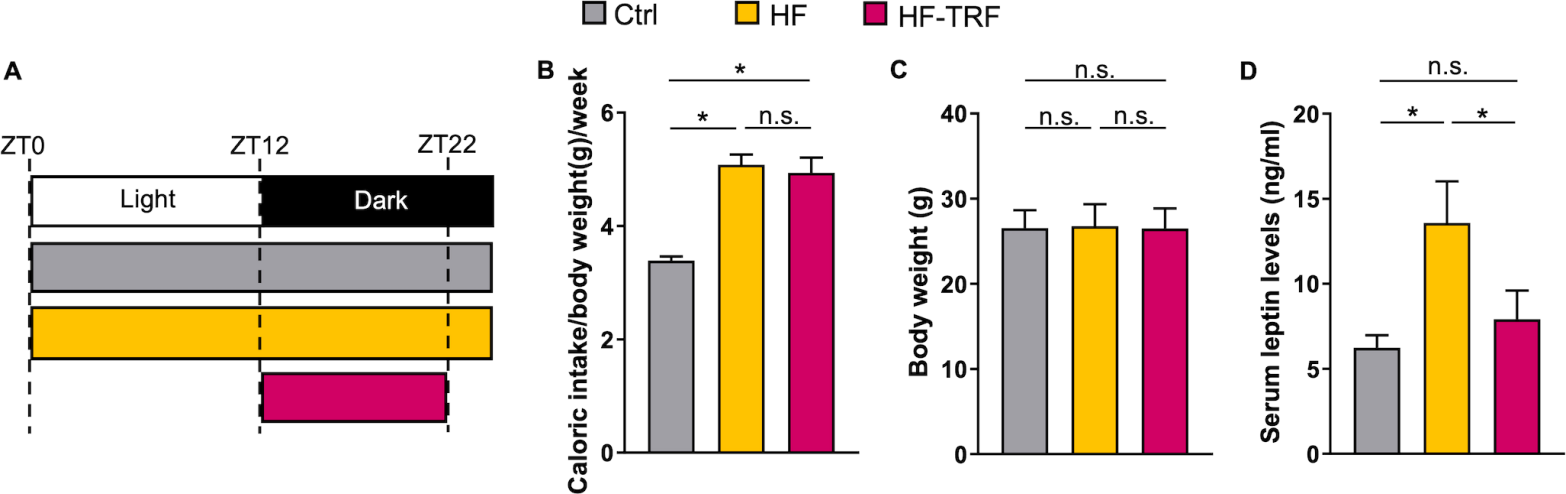
**(A)** Scheme of the feeding schedules. Mice were kept under 12 hour light/12 hour dark schedule. Control (Ctrl) group had constant access to standard chow. High-fat (HF) groups consumed high-fat containing chow either *ad libitum* (HF), or time-restricted (HF-TRF). HF-TRF mice had food access only in the first 10 hours during their active period, between ZT12-22. ZT= *Zeitgeber* time (hours after light onset). **(B)** Average caloric intake of the animals. Food intake per cage was measured three times per week and weekly averages normalized to g body mass were calculated. n_cages_= 4/group. Mean + SEM, One-way ANOVA, *Post Hoc* Fisher LSD Test, *p<0.05, n.s., not significant. **(C)** Body weight of the animals after the 4-week conditioning. n(Ctrl)=17, n(HF)=16, n(HF-TRF)=17, Mean + SD, One-way ANOVA, n.s. not significant. **(D)** Daily average of *steady-state* serum leptin levels. Blood samples of animals subjected to the feeding schedules for 4 weeks were collected at six designated time points (ZT1, 5, 9, 13, 17, 21), serum was prepared and leptin levels were measured. The daily average leptin level was calculated in each group. n=3/time point/group, Mean + SEM, One-way ANOVA, *Post Hoc* Fisher LSD Test, *p<0.05, n.s., not significant.

Male C57BL/6 mice were bred and housed in a conventional animal facility on an *ad libitum* chow diet. Light/dark periods followed a 12 hour light/12 hour dark schedule. At 60-80 days of age, animals were assigned to 3 different feeding regimens for 4 weeks: standard chow (ssniff Spezialdiäten GmbH, S8189, kJ%: fat 17%; carbohydrates 49%; protein 34%, ratios of saturated- (SFA), mono- (MUFA) and polyunsaturated (PUFA) fatty acids are 1.11:1.72:4.19) *ad libitum* fed group as a control (Ctrl), high-fat chow (ssniff Spezialdiäten GmbH, D12230, kJ%: fat 59%; carbohydrates 26%; protein 15%, SFA: MUFA: PUFA ratio is 33.55:0.75:1.37) *ad libitum* fed (HF), and high-fat chow time-restricted fed (HF-TRF) groups (**Figure 1A**). HF-TRF mice had food access only in the first 10 hours of the dark (active) phase controlled by an automated FeedTime^®^ system (TSE Systems). Animals had unlimited access to water and were kept on grid to avoid snacking and coprophagy. Weight and calorie intake of the animals were measured three times a week and the weekly average of the data was calculated. Experiments were approved by the Animal Experimentation Review Board of the Semmelweis University and the Government Office for Pest County (Hungary) (Ethical approval: PE/EA/1967-2/2017 (KA-2281)).

### 2.2 Collecting blood samples from animals subjected to the feeding schedules

As preparation of high-quality serum samples requires larger blood volumes, *steady-state* serum cytokine/adipokine levels could not be measured in the same animals in which arthritis was induced. At the end of the 4-week feeding period, 500 μl blood was collected through retroorbital sampling at a single time point from each mouse subjected to the Ctrl, HF, and HF-TRF protocols, at ZT1, 5, 9, 13, 17, and 21 for serum preparation (ZT= *Zeitgeber* time - time after light onset, ZT0-12 light phase, ZT12-24 dark phase). For identification of leukocytes, 30 μl blood was collected after tail snip. Sampling in the dark phase was performed under red light.

### 2.3 K/BxN serum-transfer arthritis

STA was induced as described previously (20). The feeding-regime was continued during the arthritis development. Arthritis severity was assessed on the 6^th^ day after arthritis induction (at the peak of the arthritis symptoms) at ZT5 by two investigators independently by measuring the ankle thickness with a spring-loaded caliper (Kroeplin) and by clinical scoring of the ankles. The subjective clinical arthritis score was obtained by visual inspection of the signs of arthritis, such as redness, swelling, and deformity. Each paw was assigned a score ranging from 1 (no signs of arthritis) to a maximum value of 10 based on the severity of the symptoms. Values measured by the two investigators were averaged and normalized to data obtained on the day of arthritis induction. To assess articular function, mice were placed on a custom-made wire grid with wire thickness and spacing identical to a regular wire cage lid. The grid was flipped upside down and the length of time the mice held on to the grid was recorded (24). This process was repeated, and the average of the two values was calculated. For further investigations, mice were terminated at ZT5 by cervical dislocation, back limbs were cut off, minced and incubated in a digesting solution [200 mM HEPES (pH 7.4), 200 μg/ml Liberase TM (Roche), and 1 mg/ml DNase I in HBSS (Gibco)] on a horizontal shaker for 1 hour at 37°C and 1400 rpm. The cell suspension was strained (40 μm filter) and centrifuged for 5 min at 500 g. Cell pellet was used in flow cytometry, whereas the supernatants were stored at -80°C and used for ELISA. Both hind limbs of each experimental animal were collected, and mean values of the left and right limbs per individual mouse were used as data included in the figures and statistics.

### 2.4 Analysis of leukocyte subsets

Blood leukocyte populations were identified as described previously (20). The absolute cell count in the digested hind limbs was determined using CountBright (Invitrogen). Cells were labeled with anti-CD45-FITC, anti-Ly6G-PE, and anti-CD11b-eFluor450 (Invitrogen) (**Supplementary Table 1**). Neutrophils were identified as CD45+, Ly6G+, CD11b+ cells, whereas the CD45+, Ly6G-, CD11b+ population referred to the monocyte/macrophage pool. For analysis of flow cytometric measurements (CytoFLEX, Beckman Coulter) the Kaluza Analysis Software (version 2.1, Beckman Coulter) was applied. Gating strategies are indicated in **Supplementary Figure 1**.

### 2.5 Leptin and IL-1ß measurements

Leptin and IL-1ß levels were determined in the supernatants of the digested hind limbs and in the serum samples using ELISA kits (R&D Systems). To prepare serum samples, blood samples of animals subjected to the same feeding schedules (Ctrl, HF, HF-TRF) for 4 weeks as mice with STA were collected at six designated time points (ZT1, 5, 9, 13, 17, 21). Daily average leptin level was calculated in each group.

### 2.6 Histological analysis

Ankles were collected, and fixed with 4% paraformaldehyde. Decalcification was carried out in Osteosoft^®^ solution. After dehydration, samples were embedded in paraffin; sagittal sections (6 µm) were stained with hematoxylin and eosin. Sections were obtained from standardized depths relative to the same joint structures across all samples. Images were taken with a Nikon Upright ECLIPSE microscope. Bone destruction, granulation tissue and swelling were analyzed.

### 2.7 Statistical analysis

Statistical analysis was performed using Statistica software version 14.0.1 (StatSoft). Statistical significance was set at p<0.05. Measurements were taken from distinct samples. Comparisons between two groups were carried out by two sample t-tests. Group differences were tested by one-way ANOVA, followed by Fisher’s LSD *Post Hoc* Test. In case of the analysis of grid-holding ability, Log-rank (Mantel-Cox) test was applied (GraphPad Prism 8.0.1).

## 3 Results

### 3.1 Symptoms of arthritis can be mitigated through time-restricted feeding

To address the impact of both food composition and timing of food intake on arthritis development, animals were divided into three experimental groups. Mice fed standard chow *ad libitum* were considered as the control group (Ctrl). Metabolic disturbance was triggered with an *ad libitum* high-fat diet (HF), whereas the HF-TRF group had food access limited to the first 10 hours of the active phase (dark period) (**Figure 1A**). Mice were conditioned to the feeding schedules for 4 weeks. HF and HF-TRF groups had similar caloric intake, which was significantly higher compared to the Ctrl group (**Figure 1B**; **Supplementary Figure 2A**). HF mice consumed relatively large fraction (24%) of the chow during the light phase (ZT0-ZT12), which significantly differed from the daytime food intake observed in the Ctrl group (17%) (**Supplementary Figure 2B**). As body weight gain can contribute to joint function deterioration and therefore exacerbation of arthritis symptoms, and even a short-time high-fat diet increases the body mass, it was important that mice had similar body weight. Our HF feeding schedule induces significantly greater weight gain compared to that observed in the Ctrl and HF-TRF animals (**Supplementary Figure 2C**). To minimize the influence of body weight, the 4-week HF *ad libitum* conditioning was started with mice with lower initial body mass, ensuring their weight matched the other two groups after the 4-week feeding schedule (**Figure 1C**). To validate the metabolic effects of the feeding protocol, we measured the *steady-state* serum leptin levels of the animals at the end of the 4-week conditioning. In the HF group, the average leptin concentration was roughly double that of the group on a standard diet, while HF-TRF feeding prevented this increase (**Figure 1D**). This suggests that even this short 4-week HF feeding significantly alters the animals’ metabolic state.

**Figure 2:**
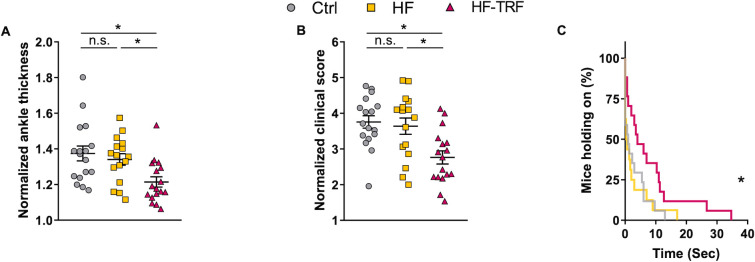
Phenotypic analysis of STA. Ankle thickness of the limbs **(A)** and clinical scoring **(B)** were normalized to values measured directly before arthritis induction (day 0). n(Ctrl)=17, n(HF)=16, n(HF-TRF)=17, Mean ± SEM, One-way ANOVA, *Post Hoc* Fisher LSD Test, *p<0.05, n.s., not significant. **(C)** Grid-holding ability of the animals. n(Ctrl)=17, n(HF)=16, n(HF-TRF)=17, Log-rank (Mantel-Cox) test, curve comparison, *p<0.05.

After conditioning to the 4-week feeding schedules, STA was induced and the feeding-regime was continued during the arthritis development. Arthritis severity was assessed on the 6^th^ day after induction by ankle thickness measurements, clinical scoring of the paws, and functional grid tests. In the HF group neither the ankle thickness nor the clinical score differed from that of Ctrl (**Figures 2A, B**). In the HF-TRF group, however, both parameters were below the levels measured in both the Ctrl and the HF animals (**Figures 2A, B**), indicating an inflammation mitigating effect of TRF. Grid-holding ability showed similar differences as the phenotypic analysis; the HF and Ctrl groups did not differ (**Figure 2C**), and the performance of HF-TRF mice was better compared to both other groups (**Figure 2C**), again suggesting an anti-inflammatory effect of TRF.

### 3.2 High-fat diet enhances, whereas time-restricted feeding reduces synovial leukocyte infiltration and levels of inflammatory mediators

**Figure 3:**
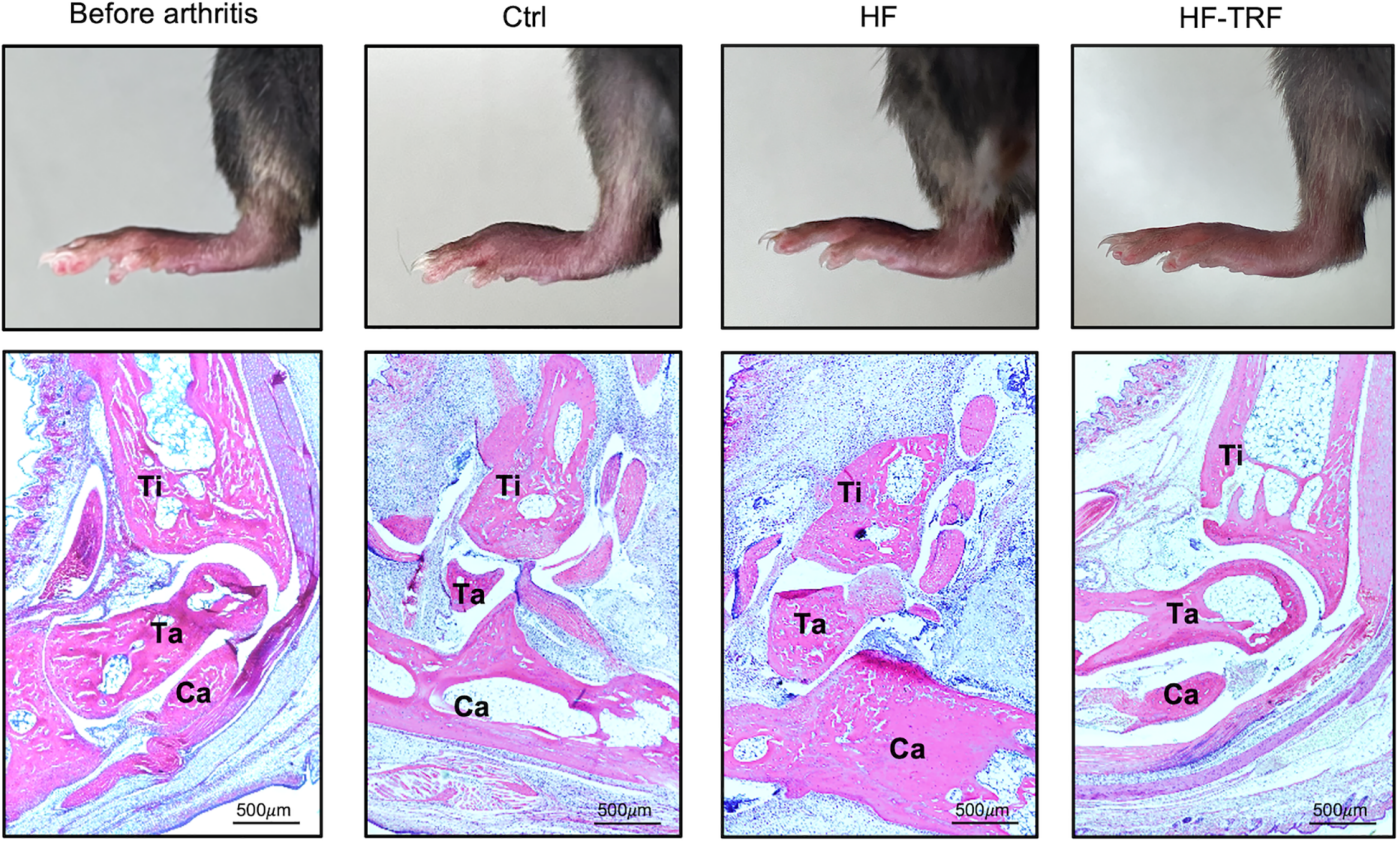
Histological analysis of the ankles. Hind limb images (upper panels) and hematoxylin-eosin stainings (lower panels) of section from the Ctrl group before arthritis induction, and of sections from the Ctrl, HF, and HF-TRF groups on the 6th day after arthritis induction. Bones were labeled in the images: Ti, Tibia; Ta, Talus and Ca, Calcaneus.

To further examine the inflammation, histological analysis was conducted. Expanded edema, increased pannus formation, bone erosion and leukocyte infiltration were observed in the joints of HF mice when compared to the Ctrl group (**Figure 3**; **Supplementary Figure 3**). These markers were, however, less pronounced in HF-TRF ankles, confirming the alleviating effect of TRF on STA development (**Figure 3**; **Supplementary Figure 3**). Based on the analysis of the nuclear segmentation of the cells, increased neutrophil infiltration was observed in HF mice compared to the Ctrl, which was significantly mitigated in the HF-TRF group (**Supplementary Figure 4**).

**Figure 4:**
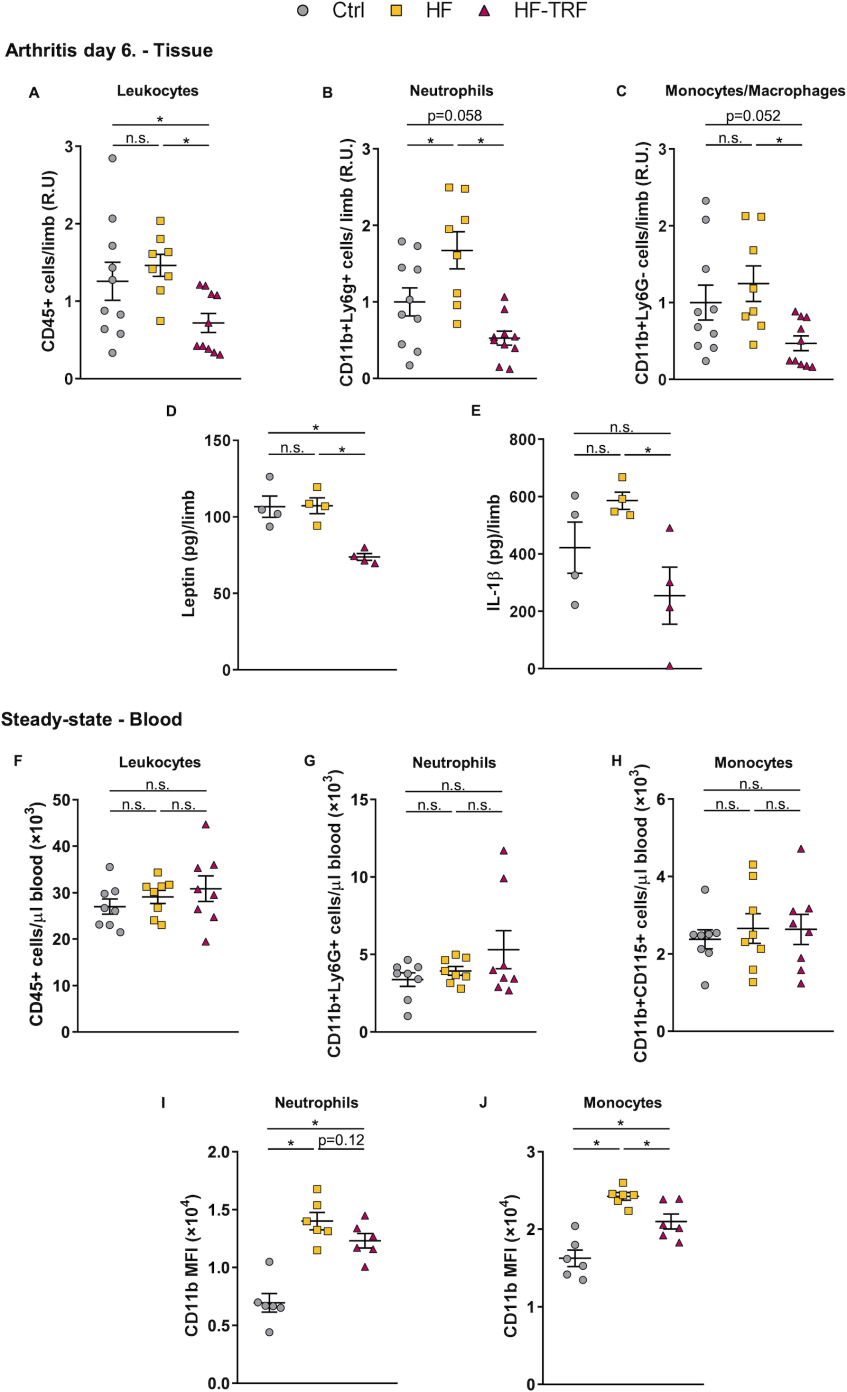
Analysis of leukocytes and cytokines in arthritic limbs, and of *steady-state* blood leukocyte subsets. **(A)** Leukocyte (CD45+), **(B)** neutrophil (CD45+, CD11b+, Ly6G+), and **(C)** monocyte/macrophage (CD45+, CD11b+, Ly6G-) counts in the digested limb samples at ZT5. In distinct experiments, values were normalized to average cell counts measured in the control samples. n(Ctrl)=10, n(HF)=8, n(HF-TRF)=10, Mean ± SEM, One-way ANOVA, *Post Hoc* Fisher LSD Test, *p<0.05, n.s.: not significant. R.U. relative unit. Synovial leptin **(D)** and IL-1β **(E)** levels. n=4/group, Mean ± SEM, One-way ANOVA, *Post Hoc* Fisher LSD Test, *p<0.05, n.s.: not significant. **(F)** Leukocyte (CD45+), **(G)** neutrophil (CD45+, Ly6G+), and **(H)** monocyte (CD45+, CD11b+,CD115+) counts in blood at ZT5 in *steady-state*. **(I, J)** Daily average CD11b expression on circulating neutrophils **(I)** and monocytes **(J)** in *steady-state*. Blood samples of animals subjected to the feeding schedules for 4 weeks were collected at six designated time points (ZT1, 5, 9, 13, 17, 21) and subsequently analyzed. The daily average CD11b level was calculated in each group. n=3/time point/group, Mean ± SEM, One-way ANOVA, *Post Hoc* Fisher LSD Test, *p<0.05, n.s.: not significant. MFI: mean fluorescent intensity.

Enhanced migration of leukocytes to the synovium could contribute to aggravation of inflammation; therefore, the leukocyte count and composition in the arthritic limbs were further investigated. On the 6^th^ day after arthritis induction, synovial leukocyte count in HF mice was slightly elevated compared to the Ctrl group, whereas in HF-TRF animals, it was significantly lower than in both the Ctrl and HF groups (**Figure 4A**). Among leukocytes, the neutrophil population in the HF mice was almost twice as high as that in the control animals, and a similar tendency was detected in the monocyte/macrophage population (**Figures 4B, C**). In the HF-TRF group both cell populations were diminished when compared to HF animals (**Figures 4B, C**), indicating that the reduced myeloid infiltration might contribute to the decreased inflammation observed in the HF-TRF group.

Next, we investigated how TRF affects cytokine production. In contrast to cellular data, no difference in leptin (**Figure 4D**) or IL-1β (**Figure 4E**) in the arthritic limbs was detected between the HF and Ctrl groups. In the HF-TRF animals, however, levels of leptin was significantly decreased (**Figure 4D**), parallel with the reduction of synovial IL-1β (**Figure 4E**) when compared to HF animals.

As we found elevated serum leptin levels and neutrophil infiltration in HF animals, and leptin is known to trigger leukocyte activation, we hypothesized, that leukocytes are already primed before STA induction. Therefore, the effect of feeding schedules on the activation state of myeloid cells in blood was investigated in the *steady-state*. After the 4-week feeding protocol, the total leukocyte, neutrophil, and monocyte counts did not differ among the three groups (**Figures 4F, G, H**). However, CD11b expression on both neutrophils and monocytes was increased in HF animals, whereas HF-TRF tended to decrease its expression on neutrophils and significantly reduced its levels on monocytes (**Figures 4I, J**). These data suggest that the HF diet had a priming effect on the myeloid cells which might contribute to the elevated migratory capacity of the cells in the STA model.

## 4 Discussion

Several clinical and experimental data show that unhealthy eating could be a risk factor of the development or exacerbation of autoimmune inflammatory diseases. In this study, we showed that even a relatively short period of high-fat diet significantly exacerbated the symptoms of joint inflammation. Limiting the feeding regime to only four weeks allowed us to primarily focus on the effects of metabolic changes on inflammation. A longer high-fat diet would lead to significant obesity, as well as leptin resistance, which we aimed to avoid in this study. Four weeks were sufficient to induce metabolic changes (reflected *e.g.* by elevated leptin levels) without causing excessive metabolic syndrome. A high-fat diet can aggravate arthritis through several mechanisms. It promotes obesity which enhances the mechanical stress on joints, particularly exacerbating osteoarthritis (25). However, to avoid the influence of the body weight on arthritis development, we used mice with similar weight in all experimental groups. Importantly, a recent paper demonstrated that HF diet worsens the symptoms of collagen-induced rheumatoid arthritis even without increasing body weight (26). Furthermore, HF diet contributes to systemic inflammation by enhancing the production of proinflammatory cytokines and adipokines in the adipose tissue (27). Fat-rich diet also disrupts lipid metabolism, leading to the accumulation of pro-inflammatory lipids in joints, which exacerbates synovial inflammation and cartilage degradation (28). Additionally, a HF diet alters gut microbiota, contributing to systemic inflammation, increasing oxidative stress and further worsening the joint tissue damage (29). It is also important to note that the fatty acid composition can specifically influence both the metabolic and immunomodulatory effects of the HF diet. Diets rich in saturated fatty acids (SFA) can promote ectopic lipid accumulation and trigger low-grade systemic inflammation, which may contribute to the onset of insulin resistance and obesity. In contrast, diets high in polyunsaturated and monounsaturated fatty acids are proposed to alleviate this inflammatory response [reviewed in (30)]. Accordingly, in our model, even a short 4-week period of high-fat diet enriched in SFA – in particular, lauric acid - significantly exacerbated the symptoms of inflammation.

Previously, dietary habits were deemed unhealthy primarily based on food composition alone, but recent studies increasingly emphasize the importance of timing of food intake for maintaining health. Growing evidence supports that timing of food consumption strongly influences the circadian clock function in both the liver and the adipose tissues, and TRF can therefore significantly strengthen the rhythm of metabolism (31, 32). Compared to HF-TRF mice, *ad libitum* fed Ctrl and HF mice consumed food in a significantly longer period of the day, which can dampen the metabolic rhythm. Additionally, HF mice eat significantly more during the inactive period, which can further weaken the metabolic rhythm compared to the Ctrl. Time-restricted feeding in animal models or time-restricted eating (TRE) in human are beneficial to mitigate obesity and lower the systemic inflammatory potential without needing caloric restriction or changes in the nutrient composition. Hatori and colleagues demonstrated that TRF without reducing caloric intake prevents metabolic diseases in mice fed a high-fat diet; it inhibits body weight gain and normalizes glucose- and lipid homeostasis (19).

Adipose tissue dysfunction is closely linked to metabolic syndrome and inflammation. In our recent study we found, that short-term TRF can enhance the rhythmic adipose tissue function and can therefore lower systemic inflammatory potential through reduction of expression of inflammatory mediators (*tnfα, nlrp3 and il-18*) and adipokines (*leptin, adipsin*) in epididymal visceral white adipose tissue (20). Besides, TRF strengthens the rhythmicity of leukocyte count in the blood and reduces the tissue accumulation of myeloid cells during the resting phase by modifying the adhesion molecule expression, which reduces the risk of tissue damage and autoinflammatory processes (20). These observations suggested that the modulation of adipose tissue through TRF contributes to the alleviation of arthritis symptoms. The large reduction of leptin levels by TRF raises the possibility, that - as a cytokine - it played an important role in regulating arthritis development in our experiments (33). According to the literature, leptin contributes to arthritis pathogenesis by affecting synoviocyte function and cytokine production, and therefore, promoting inflammation (33–35). Additionally, it increases matrix metalloproteinase (MMP) activity, leading to extracellular matrix degradation and joint destruction (36). Furthermore, by influencing immune cells and promoting angiogenesis within the synovium, leptin enhances the overall inflammatory state (34). Our results and literature data show, that TRF prevents the elevating effect of HF diet on leptin production (19, 20), which could contribute to the reduced inflammatory phenotype observed in our K/BxN serum-transfer arthritis model. Furthermore, TRF diminishes leukocyte infiltration to the synovium, and this may be partly due to a decrease in CD11b expression on monocytes. Leptin is known to trigger the expression of β2-integrins on myeloid cells (22, 23), which also supports the hypothesis that adipose tissue modulation and decrease in leptin levels might be important mediators of the TRF effect. Additionally, as *ad libitum* feeding with standard chow has been shown to induce a pro-inflammatory state compared to TRF with standard chow (20), this proinflammatory effect may explain why levels of some inflammatory markers in the HF-TRF group decreased below those of the control group in our experiments.

Rheumatoid arthritis symptoms in both humans and mice exhibit diurnal patterns, peaking during the respective inactive phases (18). Therefore, we measured the symptoms of arthritis at ZT5 in the middle of the inactive phase of mice. Acute effects of feeding during the light phase on inflammation cannot be entirely excluded in *ad libitum* fed animals, however, several lines of evidence suggest that at ZT5 it had minimal impact on our results. Although we did not precisely track the timing of food intake, all animals were observed to be asleep at ZT5 at the start of the measurements. Moreover, based on previous data and our own observations as well, *ad libitum* fed animals consume most of their chow at the very end of the active phase and the very beginning of the inactive phase, with minimal food intake occurring after ZT3 (19).

In summary, our findings suggest that even a short-term TRF may counteract the proinflammatory effect of a high-fat diet, and provide a sustainable, preventive, and non-pharmacological approach to managing RA.

A limitation of the study is that exclusively male animals were used in the experiments to minimize the potential variability introduced by hormonal fluctuations in females, such as those associated with the estrous cycle, which are known to significantly influence metabolic processes and inflammatory responses. While this approach was intended to enhance the consistency and reliability of our findings, it inherently limits the generalizability of our results, particularly given that arthritis is more prevalent in females than in males. Future studies should incorporate female animals to better understand the sex-specific mechanisms underlying arthritis and to ensure broader applicability of the findings.

Another limitation of our study is the use of the K/BxN serum-transfer arthritis model, which predominantly reflects the effector phase of rheumatoid arthritis and bypasses the early stages of disease development. As a result, the model does not allow the evaluation of key early mediators, such as those involved in the activation of adaptive immunity (*e.g.* T and B cell responses) or the production of autoantibodies. Additionally, early chemokines and cytokines, which play crucial roles in initiating joint inflammation and immune cell recruitment, could not be properly assessed.

## Data Availability

The raw data supporting the conclusions of this article will be made available by the authors, without undue reservation.
