# Supplementary material for: Time-restricted feeding alleviates arthritis symptoms augmented by high-fat diet
Source: Front Immunol. 2025 Feb 13;16:1512328. doi: 10.3389/fimmu.2025.1512328 (PMC11864949; doi:10.3389/fimmu.2025.1512328)
Supplement: Supplementary file 1 [file DataSheet1.pdf]

Supplementary Material

Supplementary Figure 1. Gating strategy of synovial fluid leukocytes

Supplementary Figure 2. Weekly and daytime caloric intake and weight gain in the different feeding groups

Supplementary Figure 3. Histological analysis of K/BxN serum-transfer arthritis

Supplementary Figure 4. Effect of the feeding schedules on tissue neutrophil accumulation

Supplementary Table 1. Antibodies for flow cytometric analysis

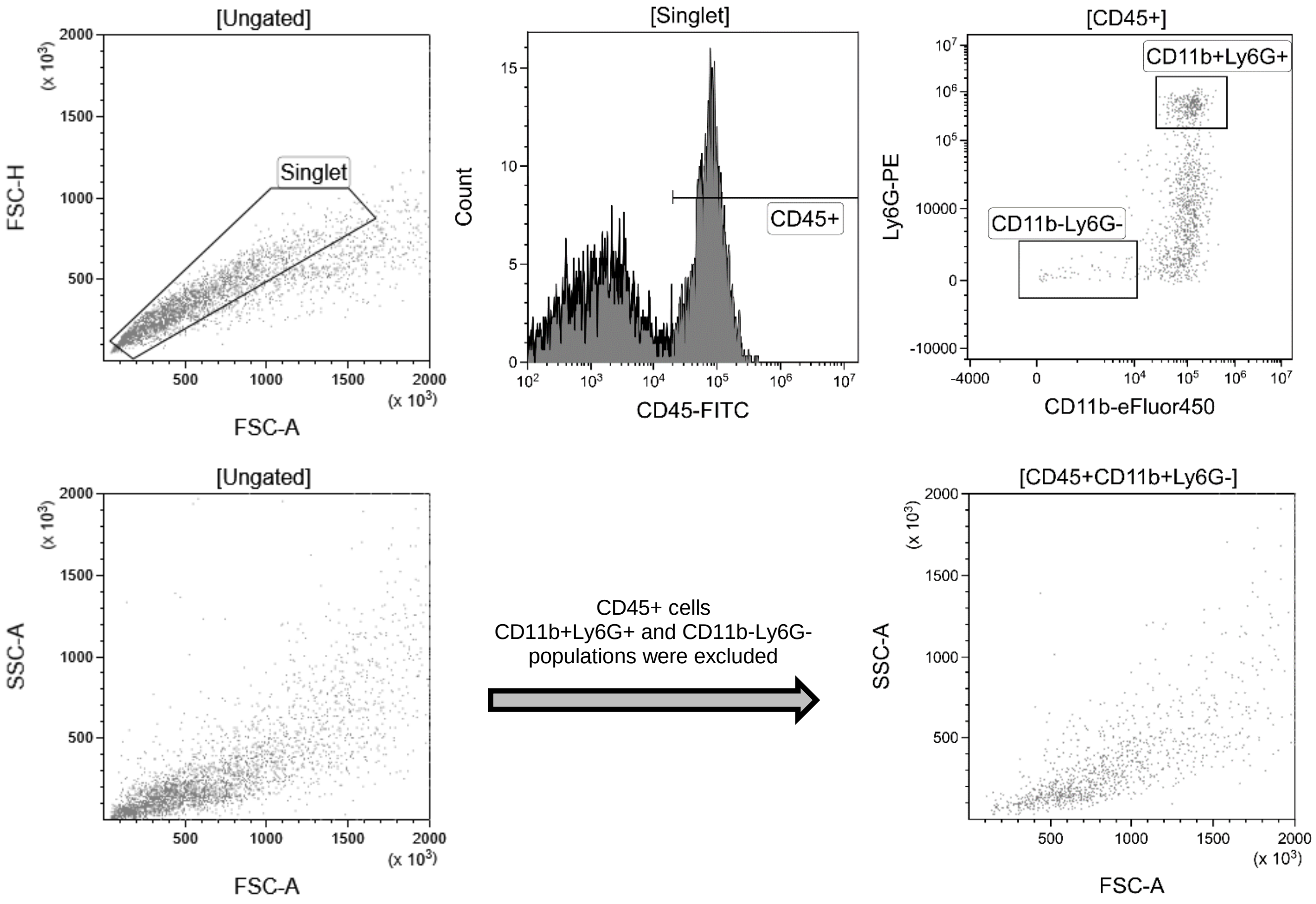

Supplementary Figure 1. Gating strategy of synovial fluid leukocytes

Singlets were gated from all events. Within the singlet cell population, leukocytes (CD45+ cells) were gated. In the CD45+ gate, neutrophils (CD45+CD11b+Ly6G+) and lymphocytes (CD11b-Ly6G-) were determined. These populations were excluded from the CD45+ cells, and then the monocyte/macrophage population (CD45+CD11b+Ly6G-) was defined.

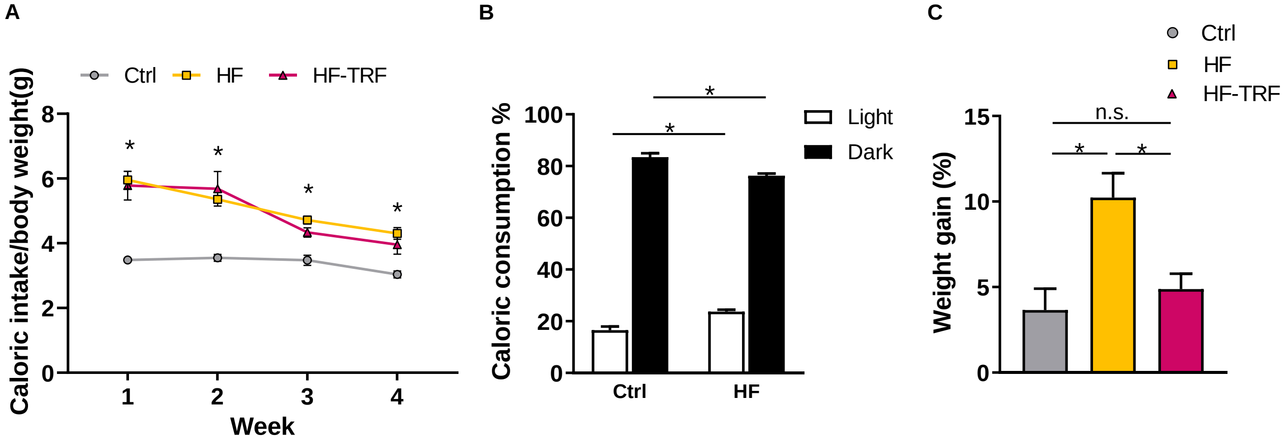

Supplementary Figure 2. Weekly and daytime caloric intake and weight gain in the different feeding groups

A) Weekly average caloric intake of the animals. Food intake per cages was measured three times per week and weekly averages normalized to g body mass were calculated. n_{cages}=4/group, Mean±SEM, Repeated measures ANOVA, significant group*time effect, *Post Hoc* Fisher LSD Test, *p<0.05. Asterisks indicate significant differences between the HF and HF-TRF groups compared to the Ctrl animals. **B)** Caloric intake ratios of the Ctrl and HF groups during the light and dark phases. n_{cages}(Ctrl)=5, n_{cages}(HF)=4, n_{days}(Ctrl)=13, n_{days}(HF)=10, Mean + SEM, Two-sample t-test, *p<0.05. **C)** Weight gain of the groups involved in the K/BxN serum-transfer arthritis experiments during the 4-week feeding schedule. n(Ctrl)=17, n(HF)=16, n(HF-TRF)=17, Mean + SEM One-way ANOVA, *Post Hoc* Fisher LSD Test, *p<0.05.

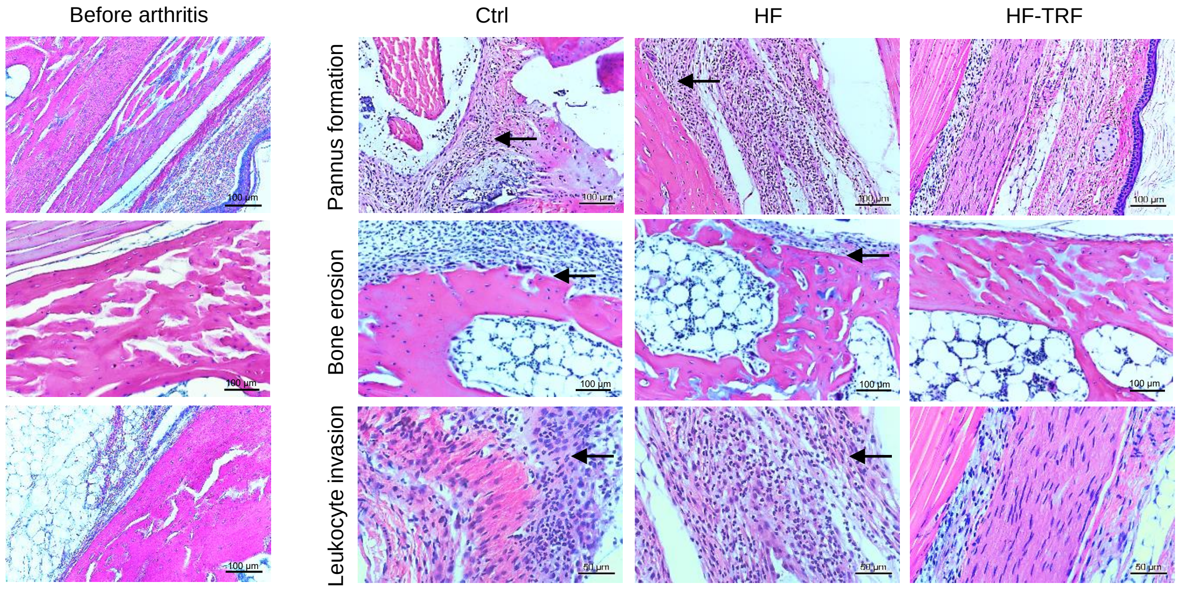

Supplementary Figure 3. Histological analysis of K/BxN serum-transfer arthritis

Arrows indicate pannus formation, bone erosion, and leukocyte invasion, respectively. Representative hematoxylin-eosin staining of samples before arthritis induction and Ctrl, HF, and HF-TRF hind limbs on the 6th day after K/BxN serum-transfer arthritis induction. As indicated, the images show pannus formation at the joint space, bone erosion at the Tibia, and leukocyte infiltration in the connective tissue region along the muscle originating from the Calcaneus.

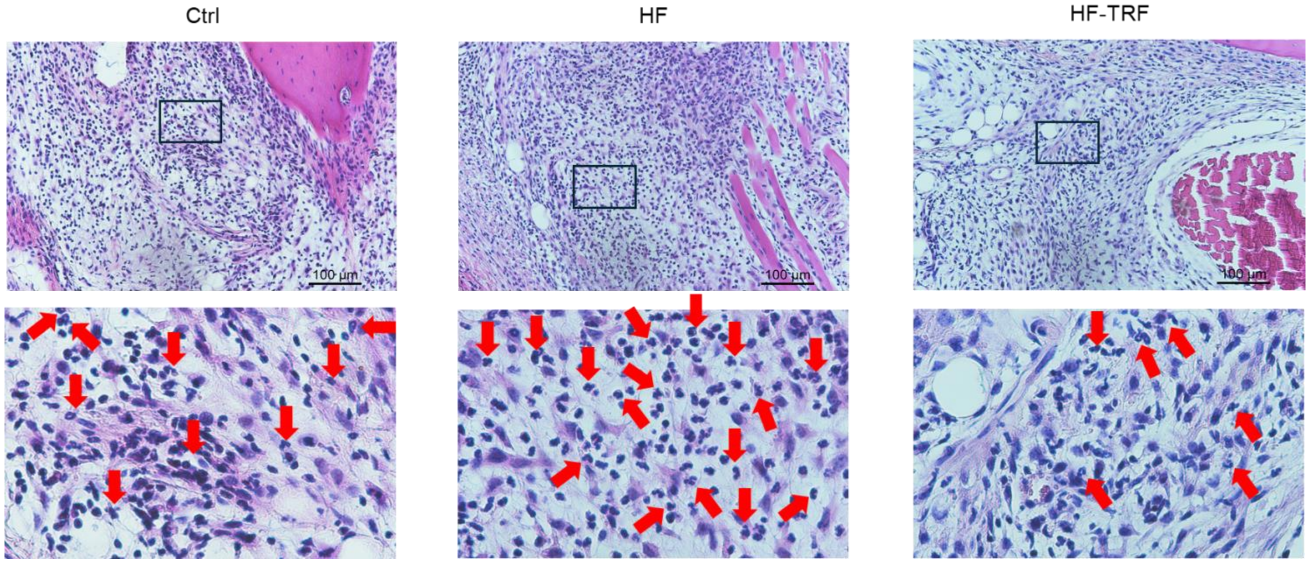

Supplementary Figure 4. Effect of the feeding schedules on tissue neutrophil accumulation

Arrows indicate cells with segmented nucleus, which are expected to be neutrophils. Representative hematoxylin-eosin staining of Ctrl, HF, and HF-TRF hind limbs on the 6th day after K/BxN serum-transfer arthritis induction. The images were taken of the connective tissue region along the muscle originating from the Calcaneus.

Supplementary Table 1. Antibodies for flow cytometric analysis.
All antibodies are products of eBioscience.

Antibody	Clone	Conjugate	Cat.no.
CD45	30-F11	FITC	11-0451
Ly-6G	1A8-Ly6g	PE	12-9668
CD11b	M1/70	eFluor 450	48-0112
CD115	AFS98	APC	17-1152
